# Excision of the primary in stage IV non-small cell lung cancer (NSCLC): A feasibility study

**DOI:** 10.1186/1749-8090-10-S1-A9

**Published:** 2015-12-16

**Authors:** Alan G Dawson, Vijay Joshi, David A Waller

**Affiliations:** 1 Department of Thoracic Surgery, Glenfield Hospital, Groby Road, Leicester, LE3 9QP, UK

## Background/Introduction

Benefit from resection of the primary tumour in stage IV renal and breast cancer has been demonstrated. This has never been shown in patients with stage IV lung cancer.

## Aims/Objectives

To establish the feasibility of a trial in NSCLC we assessed the proportion of stage IV patients in whom video-assisted thoracoscopic (VAT) wedge resection of the primary lesion would be possible.

## Method

A prospective lung cancer database was analysed to identify patients with stage IV lung cancer. Inclusion criteria included: WHO performance status of 0-2 and histologically confirmed NSCLC. Patients with cerebral metastases were excluded. The images of these patients were independently reviewed by two surgeons to determine the suitability for a VATS wedge resection of the primary lesion. Areas of discrepancy were resolved by a third senior reviewer.

## Results

Over a 14-month period, 893 patients with stage IV lung cancer were identified. A sample of 300 consecutive patients (34%) with a performance status of 0-2 were analysed of which 30 were excluded as they did not fulfil the primary criteria. Thirty-six (16%) of the remaining 230 had histological confirmation and no cerebral metastases. A further 23 patients were excluded due to the size or location of the primary. Thirteen patients (5.6%): median age 70 years (IQR: 64-76 years) were found to be suitable for a VATS wedge resection and the most common cell type was adenocarcinoma (46%).

## Discussion/Conclusion

A proposed trial of resecting the primary in stage IV NSCLC would be challenging as a busy unit would only have one candidate per month. This data will guide the design of a future multicentre trial.

